# *Recta Ratio Agibilium* in a medical context: the role of virtue in the physician-patient relationship

**DOI:** 10.1186/s13010-018-0062-3

**Published:** 2018-07-06

**Authors:** Helena M. Olivieri

**Affiliations:** Bioethics Institute, Loyola Marymount University, Los Angeles, CA USA

**Keywords:** Virtue ethics, Edmund Pellegrino, Internal morality of medicine, Physician-patient relationship, Principlism

## Abstract

Acting for the good of the patient is the most fundamental and universally acknowledged principle of medical ethics. However, given the complexity of modern medicine as well as the moral fragmentation of contemporary society, determining the good is far from simple. In his philosophy of medicine, Edmund Pellegrino develops a conception of the good that is derived from the internal morality of medicine via the physician-patient relationship. It is through this healing relationship that rights, duties, and privileges are defined for both physicians and patients. Moreover, this relationship determines the characteristics or virtues that are necessary to engage in the medical *telos*. This paper addresses the role of the moral virtues in clinical medicine and the physician-patient relationship. First, it provides a brief background of the Aristotelian foundations of virtue-ethics. Second, it delves into Pellegrino’s philosophy of medicine understood as a practice oriented towards a teleological goal. Third, it relates the *telos* of medicine to the notion of the medical community as a fundamentally moral community. Finally, it concludes with a section that creates a dialogue between virtue ethics and principlism.

## Background

*Recta Ratio Agibilium* is a Latin phrase articulated by Thomas Aquinas meaning “the right reasoning in acting” [1]. The phrase encompasses Aquinas’s notion of prudence, the capstone virtue, which provides order among the intellectual and moral virtues, while also providing for a right balance between means and good ends. With regards to clinical medicine, “the right reasoning in acting” applies to a physician’s ability in ordering scientific facts, external goods, and moral virtues in a way that prioritizes the good of the patient. The virtuous physician will be one who habitually chooses the right thing in concrete clinical situations. The integrity of the physician patient relationship is, thus, rooted in the physician’s ability to virtuously discern “the right reasoning in acting” in specific cases. Based on Edmund Pellegrino’s *The Virtues of the Medical Practice*, this paper will address the role of the moral virtues in clinical medicine and the physician-patient relationship. First, I will provide a brief background of the Aristotelian foundations of virtue-ethics. Second, I will delve into Pellegrino’s philosophy of medicine understood as a practice oriented towards a teleological goal. Third, I will relate the *telos* of medicine to the notion of the medical community as a fundamentally moral community. Finally, I will conclude with a section that creates a dialogue between virtue ethics and principlism.

## Aristotelian foundations of virtue-ethics

According to Aristotle, all things may be understood in terms of their natural functions and their proper goals. The human being itself has a specific nature, which requires certain aims and goals. It is based on these aims and goals intrinsic to the nature of the human being that directs man towards a certain *telos*. For Aristotle this *telos* is happiness, rooted in “being well and doing well in being well, of man’s being well-favored himself and in relation to the divine” [2]. The virtues are the fundamental character qualities that enable an individual to move towards *eudemonia*, the *telos.* Thus, the good life for man is one that is complete and lived at its best, and the virtues are the habitually defined excellences in character that enable man to do his work well. This means virtue entails characteristic and rational choice and action. Thus, for Aristotle the inquiry into virtue is “not in order to know what excellence is, but in order to *become* good” [3]. The purpose of ethics and philosophy is for the practical use of teaching how to live a good life.

Postmedieval eras marked a period of philosophical transformation. Distrust in metaphysics as a consequence of scientific development resulted in the dissolution of teleological theories. Teleology was replaced with utilitarian, deontological, and rights theories, among many others, each of which used the notion of virtue in various ways. As of late, there has been a revival of virtue ethics theories as well of rejustifications of Aristotelian-Thomistic traditions. Pellegrino in *The Virtues in Medical Practice* articulates a virtue theory in medical practice from the Aristotelian and Thomistic perspective [1]. His aim is to relate the virtues of medicine as a practice to the ends of medicine.

Asides from the obvious influence of Aristotle and Aquinas, Pellegrino’s work is also indebted to Alasdair MacIntyre’s notions of practice and internal good. In *After Virtue*, MacIntyre responds to the reality of the disarray of contemporary moral philosophy and morality [2]. Given societal pluralism, MacIntyre sets out to articulate a response to the criticism that there is no singular core conception of virtue. The basis of his development of a core conception of virtue is the notion of “practice.” He defines practice as a socially constructed activity, which strives for a certain *telos*. Individuals involved in the practice attend to the *telos* by achieving the internal goods of the practice. The internal goods determine the characteristics of the agents engaged in the *telos*. And, likewise, the human quality of virtue enables individuals to attend to the internal goods. However, the virtues of the practice remain undetermined until the *telos* is determined.

## Philosophy of medicine as teleological

In a concrete way, Pellegrino approaches medicine as a practice, the virtues of which remain undetermined without the clarification of the *telos*. Similar to MacIntyre, Pellegrino recognizes the moral instability of a pluralistic society. He notes that because society is constantly evolving, the internal morality of medicine is also changing. In the absence of the *telos*, external social change facilitates uncritical alterations of the medical practice. This is problematic not only because cultural morality is constantly changing, but also because unreflective medicine that conforms to the whims of societal mores may be quite dangerous (i.e. Nazi experimentation, torture of political prisoners, etc.) [1]. Pluralism, however, should not be viewed as a detriment that erodes the moral basis of cultural presuppositions; rather, it should be recognized as an opportunity to reestablished our most cherished societal values. Given the complexities of pluralism, it appears that determining the *telos* of medicine could be particularly difficult. Thus, rather than looking to an external philosophy that provides rules that shape the goods of medicine, Pellegrino suggests an alternate approach. In order to develop a notion of the *telos*, he proposes that we look at the internal morality of medicine via the physician-patient relationship.

In *A Philosophical Basis of Medical Practice*, Pellegrino articulates that medicine is fundamentally a “distinct intermediate discipline” between art and science [4]. While medicine is derived from physics, chemistry, and biology, it cannot be reduced purely to science. Likewise, medicine is obviously not a fine art, however, it does share with the arts the essence of productive reasoning for practical ends. Medicine requires competency in theoretical scientific knowledge and creative abilities in applying knowledge to individuals, thus, requiring appropriation between theory and practice through the use of prudence. Pellegrino, therefore, describes medicine as the “most scientific of the humanities and the most humane of the sciences” [4]. This special character of the medical practice, the distinct merging between art and science, is required because of the physician-patient relationship. The moral force of medicine is specifically derived from the clinical relationship because man cannot be understood merely in mechanical terms. Rather, medicine is defined by the fact that physician and patient enter into a healing relationship [4]. Thus, it is through this healing relationship that the rights, duties, and privileges are defined for both physicians and patients. Moreover, it is through the clinical context that the internal morality and the *telos* of medicine are derived.

Simply put, the *telos* of medicine is the good of the patient. In *For the Patient’s Good*, Pellegrino articulates that the good of the patient is a discrete kind of good that is specific to a “particular existential circumstance” of the individual patient [5]. That “particular existential circumstance” is the reality of being ill and needing the help of others to recover from or live with illness. The good of the patient is a multifaceted concept, which Pellegrino describes as possessing four major components [5]. The first and most important component of the patient good is the patient’s notion of the ultimate good. This notion gives context to the meaning of all human life as well as the patient’s life, from his or her own perspective. Thus, this notion constitutes the patient’s ultimate standards for his or her life choices, and also provides the fundamental groundwork for clinical decision. The second facet of the patient good incorporates the recognition of what, from the Aristotelian perspective, makes humans unique as a species- that is our ability to use reason. The second facet of the good of the patient, thus, looks at the whole of the human person who has the irreducible and uniquely human quality of choice through the use of reason. A third conception of the patient’s good must incorporate the patient’s own perception of his or her good, given the context of the patient’s life situation and his or her value system. Thus, when the patient is competent, he is to be allowed to make quality of life judgments that are consistent with his value and belief system. The final aspect of the patient good is the biomedical good.

The responsibility of the physician is to balance these components of the patient good in a way that aims at healing. The end or *telos* of medicine is a fusion of technical and moral elements of the practice that tends to the restoration or improvement of health via healing of illness or disease in a manner that prudently integrates the sciences and arts of medicine. When healing or cure is not possible, the end of medicine is care in the midst of the patient’s residual suffering.

## The *telos* of medicine and the medical moral community

Pellegrino essentially argues that the end of medicine can be best pursued in the context of a moral medical community. Much like MacIntyre, Pellegrino suggests that the goods of a practice can only be achieved when all practitioners pursue the good in concert with each other. The practice of medicine, thus, flourishes most when virtue is pursued by a community of practitioners. Today’s medicine faces a nearly irreconcilable dilemma- that is, the conflict between the primacy of altruism and the primacy of self-interest. Today’s physician is drawn to a variety of roles- that of a businessperson, scientist, corporate executive- meaning that the moral identity of the physician is pulled in numerous directions [1]. The possibilities of pluralism forces individual physicians to choose between the primacy of the covenantal physician-patient relationship and the ethos of self-interest. This dilemma not only endangers the professional ethic of medicine, but also diminishes the significance of the patient good. In order to solve this problem, Pellegrino suggests that the moral community of the profession will be required to recognize the ends of medicine in a communal way in order to prevent the erosion of the integrity of the profession [1].

Pellegrino offers three philosophical foundational reasons why medicine cannot escape being a moral community, these being: the nature of illness, the nonproprietary nature of medical knowledge, and the nature and circumstances of a professional oath. The medical relationship is one that is based on the universal phenomenon of illness. The nature of being ill is one that requires the sick person to bare their weaknesses, compromise their dignity, and reveal intimacies of body and mind. The physician-patient relationship is based on a vulnerable person voluntarily trusting a physician with their very selves. The patient is relatively powerless in this unequal relationship. Thus, because of the existential inequality of the relationship and the inescapable vulnerability of the patient, the profession of medicine has both a technical and moral claim on all physicians involved in the practice. The medical community must also be a moral community because medical knowledge has a nonproprietary nature. Certain obligations accompany medical knowledge as a result of the physician’s covenant with society. Medical knowledge is not personal private property to be utilized for personal gain; rather the physician is to be the steward of medical knowledge for the good of the patient. The professional oath of medicine, which is taken at graduation, acts as a covenant with society. The public “profession” marks the physician’s entrance into the profession. The oath is the physician’s announcement to society that he or she understands the “gravity of his or her calling” and promises to be competent and dedicated to the good of his or her patients [1].

Today’s medicine possesses two ethical conceptions of the role of the physician. One focuses on individualism, self-interest, isolation from community, and uncritical accommodations of societal and patient demands. The other ethical conception of medicine identifies that medicine is in fact a moral community, requiring doctors to transcend self-interest and political forces. Advocating the second conception, Pellegrino argues that because of the nature of illness, the nature of medical knowledge, and the oath of the profession, physicians have a collective responsibility to resist the self-interest that accompanies the medical market system. Rather, collectively, physicians as a moral community have a primary obligation to advocate for their patients.

The moral medical community is, thus, a community of physicians who voluntarily assume the duties and obligations that are associated with both the nature and ends of medicine. In order to uphold the nature of medicine and attain the ends of medical practice, individual physicians and the community as a whole must be committed to the principles of medical ethics. Virtues are the character traits that bestow individual agents with the ability to order the principles and achieve the medical *telos*.

## Virtue ethics and principlism

In a pluralistic society, there are obvious limitations of a system of ethics that is solely based on virtue. Given the heterogeneity of society, it becomes difficult to define a *telos* that is not vague. Even when the *telos* may be clearly defined, for example in the case of medicine, there is an inherent circularity to virtue ethics. A morally good act is defined by what a virtuous person would do and a virtuous person is one who does good acts. Thus, *telos* or no *telos*, there seems to be a need for specificity and a starting place. Moreover, when confronted with difficult dilemmas, a virtue-based ethics fails to provide concrete action guides. In order to avoid subjectivism, ethicists turn to principlism.

Principlism, however, also has limitations. The use of principle-based ethics has a tendency to result in a formulaic and technical application of rules, which arguably deemphasizes the role of the character of the agent. Principles are, by definition, general statements that guide a person to moral actions. However, principles themselves do not cultivate good decision-making processes. Thus, because the selection, interpretation, ordering, and application of principles are largely based on an individual’s character, it becomes clear that an ethics solely based on principles is inadequate.

Pellegrino, thus, argues that neither virtue-based ethics alone nor principle-based ethics alone are sufficient foundations for medical ethics [1]. Rather, both virtue-based ethics and principle-based ethics must be integrated in a way that cultivates the moral outlook of the medical community. Both character and principles are essential concepts in the conversation of medical ethics. This is because the medical agent is required to “[interpret] the principles, [select] the ones to apply or ignore, [put] them in an order of priority, and [shape] them in accord with his life history and current life situation” [1].

This is where Thomas Aquinas’s notion of prudence, *recta ratio agibilium*, or the “right reason in acting,” comes into play in the medical context. Moral decisions require principles because principles act as benchmarks, but general abstract principles are, ultimately, grounded in the reality of time, space, place, and persons. Thus, moral decision-making is not solely about the principle itself, rather it is about the circumstances and ends at which the principle is aimed. Consequentially, principles need to be ordered. The virtue of prudence or “the right reason in acting” enables us to discern the right and good ordering of principles in concrete scenarios. In medical practice, more times than not, the application of principles to real situations is difficult and unclear. The prudent physician is, thus, one who is capable of habitually ordering facts and principles in a way that prioritizes the good of the patient. Through the notion of prudence, virtue-based ethics, bridges abstract principles and duties to the concrete circumstances of individual lives.

Prudence not only provides order for the principles, but also provides order for other virtues. Moreover, the prudent physician is capable of relating virtues to principles. Through virtue, a physician goes beyond acting according to the right principles, rather she strives for excellence – for the “fulfillment of the full implications of the spirit of the principle” [1]. By understanding virtues like compassion, wisdom, courage, and justice, the physician can discern the “deeper and genuine meaning of principles” in relation to a specific situation [1]. Thus, there is a dialogue between virtue ethics and principlism that enriches both the virtues and the principles. Pellegrino articulates eight virtues that have spanned medical history that are required for the healing ends of the doctor-patient relationship, these being: fidelity to trust, compassion, prudence, justice, fortitude, temperance, integrity, and self-effacement.

As an example of the relationship between principlism and virtue ethics, I will argue that the virtues of compassion, prudence, and justice may provide specificity for the principles of beneficence and justice. Generally, compassion may be understood a habitual disposition that enriches the *telos*. In a clinical context, compassion can be most clearly defined as a character trait that enables a physician to contextualize that *this* experience is unique for *this* patient. Because the physician is able to internalize the unique context of his patient’s suffering, he, in a way, cosuffers with the patient. The physician-patient relationship is, thus, a real relationship – one that is based in the “story” of the patient’s illness. Compassion allows the relationship to resemble a friendship. However, the relationship is different from a friendship because there is an obvious distinct intellectual component that the virtue of compassion requires. The intellectual component of compassion consists of assisting the patient with her assessment of the balancing between her ultimate good and what the medical good can offer. The virtue of compassion, thus, helps the physician align the human goods that are unique to the patient with the medical goods associated with the patient’s unique predicament. This allows a physician to discern and understand the patient’s whole predicament and respond appropriately and prudently.

As mentioned above, prudence is the practical wisdom that enables individuals to discern which means are most appropriate for achieving the ultimate good. By interacting with the virtue of compassion, prudence informs the agent of the “right way of acting” based on the ordering the technical and moral goods involved in the practice. In relation to compassion, prudence balances and provides the intermediate between impersonal objectivity and clouded over-involvement.

Justice is the only virtue that is also a principle. Justice as a principle requires that we give each what is their due. Justice as a virtue is the habituation of giving others their due. Simply being human and living in concert with others in society requires each individual to be committed to the common good and to give others their due. Simultaneously, it is important to recognize that the common good has an individual dimension as well as a communal dimension. Thus, in a clinical sense, justice requires physicians to work benevolently towards the individual patient’s good. The focus of clinical justice must be the good of the patient as the ends, rather than any form of self-interest as the ends. Contemporary justice in our society, however, is essentially a form of depersonalized reciprocity. We owe others their due because we expect to be given our due and because we want to avoid unjust claims of others. Justice primarily becomes an obligation-based system that promotes self-interest. A virtue-ethics approach to justice suggests that “justice has its deepest roots in love”- that is love of others, rather than love of self [1]. The claim of justice is one that recognizes a fraternal nature of a community based in compassion and care. Thus, ways of demonstrating habitual acts of justice is through concrete acts of beneficence towards specific persons.

From the principlist approach, Beauchamp and Childress identify Aristotle’s principle of formal equality as the basis of justice. The principle states, “equals must be treated equally, and unequals must be treated unequally” [6]. Beauchamp and Childress identify this principle as “formal” because there is little specificity. I would argue that the principle of beneficence and the virtues of compassion, prudence, and justice offer some specificity. Compassion informs the physician that each patient is a unique individual, who deals with a unique illness, and possess a unique understanding of his or her human good. Prudence enlightens the physician on how to balance the unique goods and dynamics of each patient. The prudent physician is the compassionate physician. Simultaneously, the virtue of justice requires the physician to give each his or her due. Thus, it becomes clear that what each patient is due is an equality that recognizes the uniqueness of his or her circumstances and being. The principle of justice is only complete when the incommunicability and good of each patient is recognized and concretely treated beneficently.

## Conclusion

Ultimately, the challenge for medicine today is to develop both theoretical and concrete connections between the virtues and the principles that aim at the patient good. I agree with Pellegrino when he argued that virtue is best conveyed via a role model or mentor. Thus, the challenge for contemporary medical institutions is to cultivate a community of physicians who are grounded in virtue and capable of sharing the richness of their practice with future generations.
